# Growth model for international academic medicine partnerships: Qualitative analysis of Ghana postgraduate Ob/Gyn training program

**DOI:** 10.1371/journal.pgph.0000546

**Published:** 2023-01-12

**Authors:** Theresa L. Rager, Melani Kekulawala, Yael Braunschweiga, Ali Samba, Tim R. B. Johnson, Frank W. J. Anderson

**Affiliations:** 1 University of Michigan Medical School, Ann Arbor, MI, United States of America; 2 Department of Health Behavior and Health Education, University of Michigan School of Public Health, Ann Arbor, MI, United States of America; 3 University of Toledo College of Medicine, Toledo, OH, United States of America; 4 Division of Primary Care and Population Health, Stanford University School of Medicine, Stanford, CA, United States of America; 5 Department of Obstetrics and Gynecology, University of Ghana, Accra, Ghana; 6 Department of Obstetrics and Gynecology, University of Michigan, Ann Arbor, MI, United States of America; University of British Columbia, UNITED KINGDOM

## Abstract

This study aims to detail the capacity strengthening process of the Ghana Ob/Gyn postgraduate training program in order to inform a model by which international academic medicine partnerships can form, grow, and effectively tackle development challenges. A qualitative analysis with grounded theory methodological approach was utilized. Convenience and purposive sampling were used to select certified Ob/Gyn training program graduates. Interviews were conducted face-to-face in in Accra, Kumasi, Cape Coast, and Tamale, Ghana between June 21 to August 20, 2017. An additional data analysis of 48 semi-structured interviews previously collected for another study were examined for factors pertinent to graduate career development. Coded data were grouped according to themes and subthemes. Emerging themes demonstrated that graduates further complete the maternal care team and facilitate collaboration amongst healthcare workers. Themes also included graduates’ pursuit of subspecialty training and research. Graduates cited the training program as key to their professional development. Graduates assume leadership roles in hospital management and operations, teaching, mentoring, interprofessional maternal care team, and knowledge-sharing. Graduates expressed eagerness to subspecialize and to advance their research training and skills. The results suggest a growth model of international academic medicine partnerships from basic obstetric training to advanced training. The model is developed for adaptability in other SSA countries and low-resource settings so that it may effectively strengthen health workforce capacity. We hope that this program can serve as a model for other partnerships in medical specialties.

## Introduction

Sustainable Development Goal (SDG) 3 aims to reduce the global maternal mortality ratio (MMR) to less than 70 per 100,000 live births by 2030 [1]. While the MMR has declined 38% worldwide, Sub-Saharan Africa (SSA) accounted for approximately 67% of maternal deaths in 2017 [2]. Increasing human resources for health is an internationally-recognized goal as outlined in SDGs 3 (Health and Well-Being), 4 (Education), 5 (Gender), and 8 (Decent Work) [3]. In order to actualize these goals, the WHO Health Workforce Department has called for health workforce education, deployment and retention strategies, operational research, and strengthening of institutional capacity and partnerships [4].

In 2017, Ghana’s MMR was estimated at 308 per 100,000 live births, compared to the global estimate of 211 and SSA estimate of 560 [5]. To reduce Ghana’s MMR, adequate staffing of district hospitals by competent obstetricians is needed [2, 6]. In 1986, the Ghana postgraduate Ob/Gyn training program began as a collaboration between health, education, and development stakeholders to address the obstetrician workforce gap [7]. The program provides training Ghanaian medical school graduates (“medical officers”) at two Ghanaian institutions to mitigate Ob/Gyns traveling abroad for training and remaining abroad to practice [7]. Since 1989, the program has trained and retained over 250 certified Ob/Gyns [8].

Countries across SSA are growing their Ob/Gyn generalist population. With more generalists available, availability of basic Ob/Gyn services increases and opportunities to expand complex, subspecialty care arise. Subspecialty training broadens the care spectrum to include women’s healthcare across the lifespan and beyond maternal health [9]. Subspecialty training programs in gynecologic oncology, urogynecology, and reproductive medicine have recently begun in SSA countries [10–13]. Similarly in Ghana, subspecialty training in family planning, urogynecology, maternal-fetal medicine, and gynecologic oncology are in development. Subspecialists are able to collaborate with generalist Ob/Gyns, district health officers, midwives, and healthcare workers to further complete the maternal care team [14]. While these flagship programs set the stage for countries to achieve comprehensive obstetric and gynecologic care, physicians ability to practice subspecialized care continues to be limited by lack of training programs across SSA.

Some gynecologic oncology programs are also incorporating research skill development into their training curriculum [15]. Research is considered one of the three pillars of academic medicine (clinical care, education, and research), but the majority of health development research is led by Global North stakeholders and bypasses local research institutions [16, 17]. Incorporating research skill development augments institutions’ and countries’ research capacity. By supporting these skills, locally-driven, contextually-relevant interventions are implemented rather than enacting protocols derived in higher-resource settings [18].

As the overarching aim of the Ghana postgraduate Ob/Gyn training program is to reduce preventable maternal mortality, detailing the capacity strengthening process could inform investments in similar long-term projects. Currently, the literature lacks models for establishing and expanding international academic medicine partnerships. Thus, the objective of this study is to create such a model based on our experience in Ghana. The specific aims are to assess 1) graduates’ leadership and collaboration roles; 2) graduates’ perceptions and pursuit of subspecialty training; 3) graduates’ attitudes toward and involvement in health research. Such evaluation may reveal opportunities for program growth, suggest a larger model of global academic medicine development, and inform development initiatives to tackle future development challenges.

## Methods

### Ethics statement

The University of Michigan Institutional Review Board-HSBS approved this study and determined that it was exempt from IRB oversight [HUM00125496], and the study was approved by the University of Ghana College of Health Sciences Ethical and Protocol Review Committee [CHS-Et/M.9C/2016-2017] and the Kwame Nkrumah University of Science and Technology College of Health Sciences Committee on Human Research, Publication and Ethics [CHRPE/AP/370/17].

### Study location

Data was collected in Ghana between June and August 2017. The data was reviewed for an additional analysis in June and July 2020. COREQ criteria were used to ensure robust qualitative methodology.

### Study design

#### Participants

Both purposive and convenience sampling were used to recruit participants. Inclusion criteria included graduation from the Ob/Gyn postgraduate training program and membership and/or fellowship in the Ghana College of Physicians and Surgeons or the West African College of Surgeons since 1989. There were no exclusion criteria for this study. A roster of the 245 certified Ob/Gyn graduates with information about location, current practice setting, gender, and contact information was obtained from the Ghana College of Physicians and Surgeons and Ghana Medical Board. The roster was reviewed and information verified via department chairs or participants. Convenience sampling occurred at the university teaching hospitals in Accra and Kumasi, where many Ob/Gyns are employed or are completing fellowship training. Verbal announcements regarding study participation were made at daily morning meetings. Interested participants were scheduled for interviews on a first-come basis. To attain a more diverse sample population, purposive sampling was later utilized to recruit Ob/Gyns working outside of the teaching hospitals, including those working in non-academic settings, rural settings or other parts of the country, and recent graduates. These Ob/Gyns were contacted via information provided on the roster and other departmental resources. Through phone calls and emails, interviewees in district and private hospitals in various regions of Ghana were recruited. MK and YB then traveled to these hospitals to conduct in-person interviews. For both sampling methodologies, department chairs, administrative assistants, chief residents and other department employees assisted with participant recruitment.

#### Interviews

Both Ghanaian and U.S. researchers (MK, YB, AS, TJ, FA) from the University of Michigan, Korle Bu Teaching Hospital, and Komfo Anokye Teaching Hospital together developed a 50-question interview guide for the purposes of a qualitative study evaluating the retention and experience of graduates [19]. Consensus for the interview guide was obtained by iterative review by the authors. If consensus was unable to be obtained, decisions were made by senior investigators FA and TJ. It was not pilot tested. The guide was centered on three topics: (1) motivations for retention, practice type and location (2) role of community, social, and economic factors in retention, and practice location consideration (3) how and why the in-country training programs shaped and strengthened obstetric capacity. Interview questions from topics 1 and 2 guided this study’s analysis of the previously-collected qualitative data and are provided in Table 1.

**Table 1:** Interview questions pertaining to graduate retention and career development. Selected questions from interview guide that contributed to this additional data analysis and model development.

• What are the responsibilities of your current role? ○ What are some of the advantages (or disadvantages) of your current role? ○ Do you feel that you have the skills to serve in your current role? (And where did you learn these skills?) • What are some of your responsibilities in administrative work? ○ How, if at all, have you shaped policies within your department? ○ How, if at all, have you shaped how emergency obstetric care is delivered? ○ Can you describe some of the responsibilities you particularly enjoy or feel have made an impact on your department? • How, if at all, do you see yourself as a leader of your patient’s healthcare team? • How, if at all, has your training as an ob/gyn enabled you to be involved in teaching? ○ Could you describe your teaching responsibilities, if any? ○ How, if at all, do you see your teaching as impacting women’s health in Ghana? • Can you provide examples of collaborations with other health professionals? ○ Can you describe your collaboration, if any, with midwives? • Could you share your perspective as a woman in Ob/Gyn in Ghana? • How, if at all, has your training as an ob/gyn enabled you to be involved in research related to women’s health? ○ Could you describe research you have done since the completion of your training program? ○ How, if at all, did your training program help shape your research interests? ○ How, if at all, did your training program help develop research skills? ○ How, if at all, do you see your research as impacting women’s health in Ghana?

A total of 48 in-depth, semi-structured interviews were conducted face-to-face at the hospitals by MK and YB between June 21—August 20, 2017. Participants were provided with an explanation of the purpose and goals of the study and informed that there was no incentive for participation. Then, verbal consent was obtained and audio recorded to conduct and record the interview for all participants. All interviews were conducted in English by 1 of 2 researchers and were audio recorded. The average interview length was 31 minutes. Repeat interviews were not conducted. Participants were recruited and interviewed from the graduate roster until data saturation was reached. Saturation was determined by MK and YB’s reflexive analysis during the interview process.

#### Analysis

Data was analyzed using a grounded theory methodological approach. Audio recordings were transcribed verbatim, de-identified, reviewed, and imported into NVivo 12 by the MK and YB. Only the researchers had access to the transcripts and were not returned to participants for comments. Field notes were not used in this analysis. Each interview transcript was coded and analyzed by TR to examine factors contributing to graduate career development. A codebook was developed for data pertaining to the research questions. Patterns emerged from the coded data and were grouped according to specific aims. Patterns and groupings were reviewed by TR and FA until concurrence was reached. Based on groupings, themes and subthemes were derived from the data. Coded items were reviewed in the original transcripts to ensure contextual integrity.

#### Reflexivity

Members of the research team maintained reflexivity throughout the study design, data collection, and analysis processes. Both Ghanaian and U.S. authors were critically involved in the study design. U.S. authors (TR, MK, YB, TJ, FA) maintained awareness of possible bias due to their prior assumptions and experience outside of Ghana. Both interviewers (MK and YB) were female, trained in conducting interviews through their graduate studies, and were completing public health and medical training at the time of the study. They are not Ghanaian and are not practicing Ob/Gyns. Participants were aware of the interviewers’ credentials. Interviewers had no prior relationship with the participants. MK and YB regularly met with Ghanaian and Ob/Gyn members of the research team throughout the data collection process to discuss themes, saturation, and contextual interpretation. Members of the research team were also mindful of potential desirability bias as interviewees were graduates of the Ob/Gyn postgraduate training program and interviewers served as representatives of the program. Authors most closely involved in data collection, transcription, coding, and analysis met closely with the wider research team to discuss emerging themes and codes thereby utilizing insights of those handling the data and those with a wider perspective and contextual insights.

## Results

A total of 48 graduates participated in the study. Of the interviewees, 12 (25%) practiced in district hospitals and 36 (75%) practiced in teaching hospitals. Geographically, 4 (8%) practiced in the Cape Coast region, 5 (10%) practiced in the Tamale region, 21 (44%) in the Kumasi region, and 18 (38%) in the Accra region.

Emerging patterns charted graduates’ current professional roles and responsibilities, and career advancement. The following themes represent these patterns by specific aims: leadership and collaboration, subspecialty training, and research.

### Theme 1: Graduates are equipped to be leaders and collaborators

#### Theme 1.1: Decision-making and management

As graduates assume leadership roles on maternal care teams, they are exercising decision-making and management skills consistent with evidence-based, modern comprehensive obstetric care.

“Because you learn first of all decision making. It is very key. It’s not just doing procedures or operations. But decision making, to arrive to do or not to do a procedure for a patient.” [22051721]

“That places you in the cadre of being a leader. So it’s not something you read, it’s something you practice. So the way you coordinate your affairs, in saving a life and all that, makes you a leader.” [11140004]

#### Theme 1.2: Operational changes

Additionally, many graduates have utilized their expertise and leadership to induce operational changes that improve care or address patient needs. These changes include projects such as updating clinical flow, creating specialty clinics, introducing new protocols, or reorganizing care teams.

“We have introduced other systems like the introduction of triage to obstetric practice that has also helped us reshape the process of care. We have also introduced an obstetrical emergency unit, which again did not exist before.” [12051913]

#### Theme 1.3: Teaching and mentorship

The majority of interviewees described a passion for teaching and mentoring medical students, junior colleagues, and other health professionals. Through such efforts, many felt that they increased the skills and development of those around them to the benefit of both their colleagues and patients. Such teaching experiences take place in teaching institutions, with patients, and in the community through outreach programs.

“You also become a resource to train other people like the nurses and the midwives that work under you and other junior colleagues that you work with. You are able to transfer some of your skills and knowledge to them. So at the end of the day, you kind of replicate yourself and you make a lot more impact than you would as a [medical officer].” [12191504]

“You get to mentor people. You think strategically, so the whole department is under you, so you need to plan as to different ways, the personnel planning, the career development of your staff, because some of them may not, they want to develop their career, but they may not know how. So they may choose a course that may not be beneficial to them. So if you get to know or they come to you, then you’ll be able to guide them as to how to do it.” [12180111]

For women Ob/Gyns, mentoring opportunities were particularly important. Interviewees were passionate about mentoring young women to encourage their pursuit of a career in obstetrics and gynecology and to increase the female representation in the field.

“I became a role model for others to say that, "Yes, it is possible, it is doable." And so after me, we had four girls joining the OB-GYN program. Three of them have completed and the other one is yet to complete and there are other ladies who also want to. When they come and they see the way I go about it, they say, "Oh, then we can do it." I say, "Yes, you can do it. So come do it".” [12180111]

“right now in this department I have seven medical officers, and five of them are females. And they are all writing the exam to go to school. So I’m proud of that, because they all keep telling me that they are doing it because they’ve seen that I’ve been able to do it. So, it’s fulfilling.” [31081301]

#### Theme 1.4: Maternal care team collaboration

When asked to describe their work with midwives, graduates praised their colleagues for their knowledge, skills, and shared collaborative practice. Obstetricians considered midwives essential members of the maternal care team and patient advocates.

“In obs and gynae we can’t do anything without our midwives…So there is no obstetric care without the midwife, and the midwives form an important part of that obstetric care team.” [12191504]

As midwives are often first line of contact, obstetricians rely on strong professional relationships and communication to coordinate care.

“Then they have our mobile phone numbers, as well, both within and without the hospital, so if there are issues, they just call you. "I have this case, what should I do?" "I have this case I want to refer" they refer them. It’s been a lot of collaboration in all that.” [12110210]

Additionally, obstetricians noted the vast knowledge and skills of midwives. Interviewees expressed immense respect and gratitude for midwives who taught them during their training.

“You should have a good relationship with them, very good, very, very good relationship with them because irrespective of where you are, some of them were midwives before you became a specialist. Some of them have seen a lot. You see. You will fine tune it, but even when you’re doing that it has to be respectfully you see, respect for them, they will respect you.” [22050324]

#### Theme 1.5: Knowledge-sharing

As leaders and collaborators, graduates are seeking and disseminating knowledge within Ghana, across SSA, and around the world. Interviewees were refreshed by and encouraged to exchange ideas with colleagues, collaborate on cases, or interact with people from different disciplines and countries.

“You are here with other colleagues so sharing ideas because like no two cases are the same so sharing ideas, learning from others, others learning from you as well because eventually you stop working, but even before you stop working you should have handed over all the knowledge that you have.” [22050324]

Obstetricians explained that innovation and creativity were fostered through knowledge sharing. By seeing how other hospitals, countries, or regions conduct care, new ideas and protocols can be implemented.

“It’s good that we do the training and collaboration…because it helps us to see what is being done elsewhere. But I think everybody must come back and then it will help keep our mothers alive. Our mortalities are very high in this country, we need to reduce it.” [12200213]

### Theme 2: Graduates are eager to subspecialize

As the population of Ob/Gyn generalists (“specialists”) increases in Ghana, there is space for Ob/Gyn specialty training (“subspecialization”). One obstetrician compared this need to the need for a general Ob/Gyn training program during the 1980s by commenting on the need to leave Ghana to undergo subspecialty training.

“The first challenge would be that after the specialization, anyone who wanted to do subspecialization had a challenge … because you don’t want to go to another continent and be asked to start afresh. … So, the fact that we don’t have subspecialist programs, we didn’t have, we are now trying to develop.” [12101912]

In considering the creation of subspecialty program, others envisioned a similar model to the general Ob/Gyn training program in which, as the subspecialist population grows, these individuals can transfer their knowledge to future trainees.

“I think that we look forward to having a little more capacity now in subspecialty areas because that is coming up and then once people are trained in the areas, they will offer help in improving the health of our women and also teach others, you know and so we, we think that we are making an impact in our country more if we learn more.” [22051721]

Importantly, graduates emphasized the impact that subspecialization could have on obstetric capacity in Ghana, primarily through the availability of more advanced, skilled care.

“I mean being an OB/GYN specialist, I think now you should be thinking about subspecialization so that we will be making so much impact in that specific specialty rather than trying to do all of OB/GYN.” [22150203]

“And we just hope that we will move [postgraduate training] further up, like you saw from the beginning …how do we really move on to the subspecialties, because we really need that because we are lacking in those aspects.” [12131406]

### Theme 3: Graduates are advancing health research capacity

Nearly all interviewees described conducting research, either previously or currently. Many graduates stated they conducted research according to their subspecialty of interest, in lieu of or complementary to formal subspecialty training. Despite conducting research, when asked about its impact, one obstetrician described a need for more research:

“Research is a bit of a challenge but the only way that we can improve our protocol and to be able to change our decisions is to have empirical data on what works for our environment. So we need to do more research on a local level, and a national level.” [22051120]

In looking at the barriers to research capacity, interviewees commented on a need for funding, protected research time, supplies, and training.

“I must say, confess that research is lagging behind the other areas, teaching and then service. But the service seems to be dominating and then teaching…There is not adequate funding for research, so some of the things we have to do ourselves.” [22180122]

“I’m involved in research work, not to the extent that I want to because of the clinical workload.” [12131406]

## Discussion

In building the postgraduate training program, medical officers were recruited to train in-country, obtain obstetric skills, and become certified as Ob/Gyn specialists capable of integrating into the health system. The novel and growing Ob/Gyn population in Ghana allows for filling of maternal care team roles and collaborations, opportunity for subspecialization, and space for conducting research. Establishing graduates as practicing Ob/Gyns allows them to flourish as leaders and collaborators. Graduates are proving to be leaders of the maternal care team by exercising decision-making and management skills to serve as team and/or department managers. As such, graduates are creating operational changes to improve protocols and establish new clinics. Through their teaching, graduates are practicing academic medicine, and thereby promoting program sustainability. Additionally, women Ob/Gyns are serving as mentors and role models capable of recruiting more women into the specialty. Our results suggest that as the number of Ob/Gyn trainees and subsequently certified Ob/Gyn practitioners increases, the demand for obstetric and gynecologic care will be met. Within this structure, midwives can service uncomplicated cases and refer complex cases so that all cases are addressed with skill and competence. By bolstering the maternal care team through leadership and collaboration, preventable maternal mortality can be reduced.

Subspecialty training is an opportunity to further complete the maternal care team and broaden the spectrum of available care to include women’s health issues across the lifespan and beyond maternal health [10]. Yet, before the advent of these subspecialization programs, generalists have conducted research in their subspecialty area of interest to advance their skills and knowledge. Despite widespread involvement in research, many graduates found research capacity lacking. Participants primarily cited limited funding, lack of protected clinical time, and few training opportunities outside of subspecialty programs. Overcoming such barriers and fostering research would bring individuals and institutions to the international stage, share knowledge applicable to other low-resource settings, and create a collaborative platform to drive SDG achievement.

**Fig 1:**
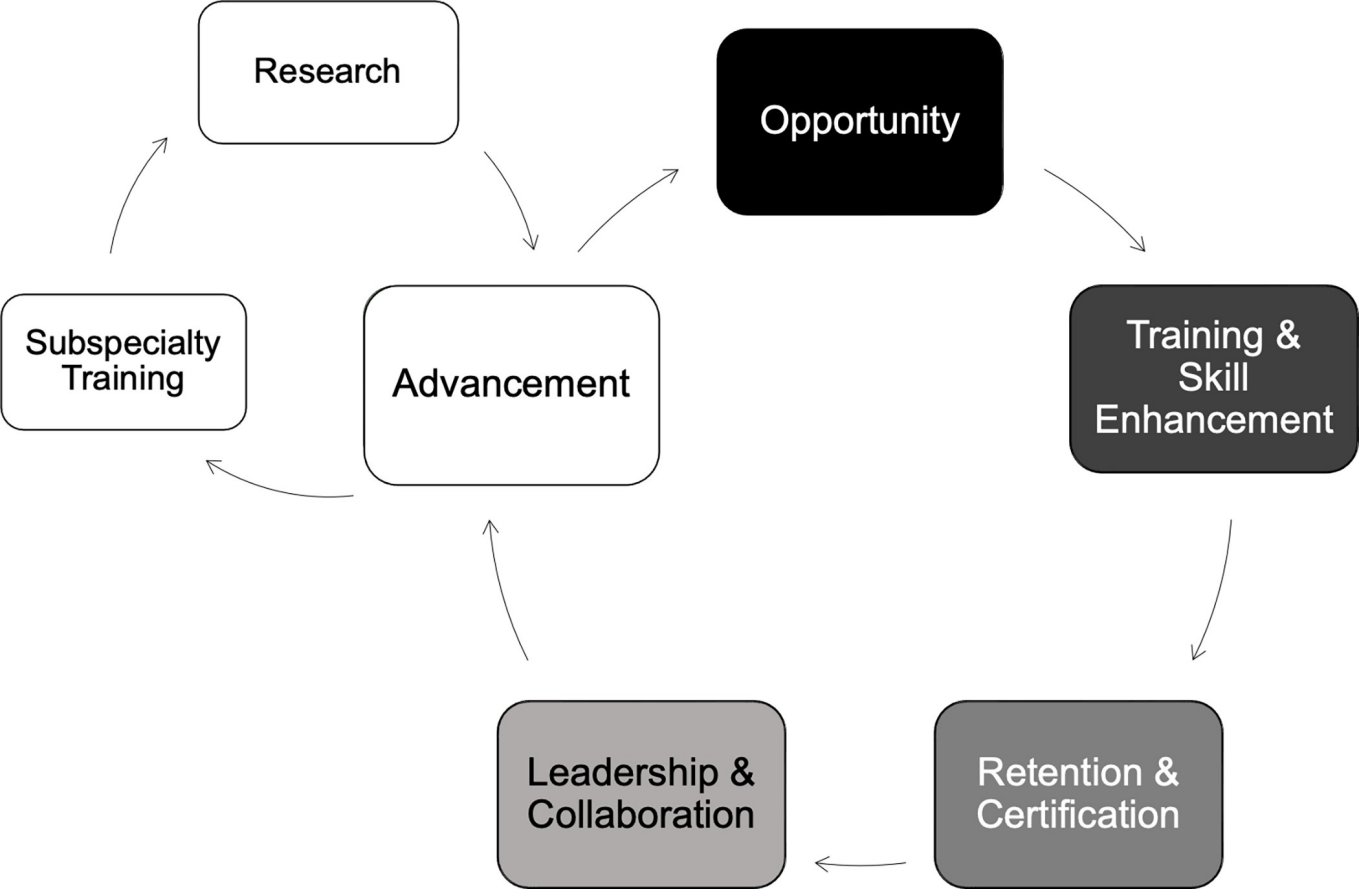
Model for postgraduate training programs aimed at health workforce capacity strengthening. The Postgraduate Training Program serves as a model by which opportunity to expand health workforce capacity is identified, training and development programs are established, graduates are retained through job creation and certified through national bodies, graduates can assume leader and collaborator roles, and then advance their careers and field through research and subspecialty training.

The results suggest a model by which postgraduate training programs strengthen the healthcare workforce, expand the specialty, and increase national capacity (Fig 1). According to the model, an opportunity to increase capacity must be first identified. Here, the postgraduate training program discovered medical officers were eager to specialize in Ghana but lacked the opportunity to advance their careers within the country [7]. Recognizing this opportunity created an environment to increase Ghana’s obstetric capacity [20]. Now, an analogous opportunity presents itself in which Ob/Gyn specialists are eager to complete subspecialization training to expand Ob/Gyn care within Ghana. In order for SSA medical institutions to actualize the three pillars of academic medicine, Ob/Gyn programs need to integrate subspecialty and research training and practicums into their curriculums. By creating an environment that allows for such advancement, comprehensive women’s health services can become more widely available and contribute to improving women and child health outcomes.

**Fig 2:**
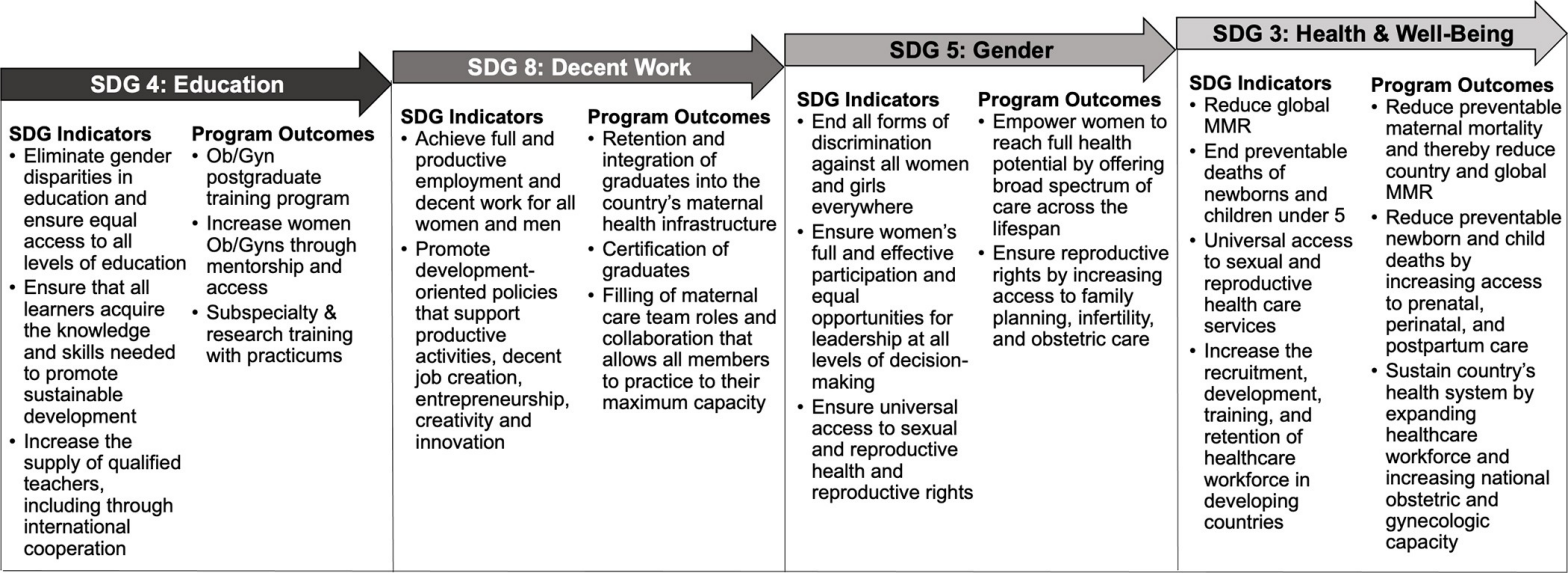
Sustainable development goals and targets addressed through ob/gyn postgraduate training program. Creating education opportunity (SDG 4) lays the foundation to promote decent work for graduates (SDG 8), leading to gender equity for both graduates and patients (SDG 5), and ultimately improves health and well-being, particularly maternal, newborn, and child health (SDG 3) [3].

This proposed model is represented as a cycle because opportunities continue to arise with ongoing development. Initial efforts were aimed at creating Ob/Gyn specialists, which we have demonstrated led to the opportunity for subspecialists and research capacity. Subsequent efforts to expand subspecialty and research training will conceivably create more opportunities for advancement as professionals continue training and further develop expertise in subspecialties and beyond. This evolution of obstetrics and gynecology practice from basic care to subspecialization and research sets the stage for countries to enact comprehensive care, optimize women’s health, and achieve the SDGs (Fig 2).

Processes, like postgraduate and subspecialty training, are successful if there is an initial investment and partnership that creates space for the process to exist, be self-sustained, and be high impact. Building programs allows infrastructure for retention, improved healthcare quality and access, a broader spectrum of care, and research platform. Many development initiatives attempt to fill gaps with projects because processes for comprehensive, national approaches are not in place. Here, we suggest a model for establishment and growth of long-term, sustainable programs that mitigate development gaps that necessitate filling by international aid. When considered as prospective, gap-mitigation efforts rather than a retrospective tackling of development challenges, training programs can truly avert preventable maternal mortality and improve women’s health.

### Limitations

There were a few limitations to this additional data analysis. Despite the sample being larger and more diverse than previous studies, there were few women in the sample (n = 4, 8%), narrowing our understanding of women’s perspective. Generalizability of our findings may be limited by unequal gender and geographic distributions. While the study is specific to Ghana, we believe the overarching themes and presented model may be applicable to other low-resource or SSA settings. Moreover, subspecialty training and research development are rapidly evolving areas within Ghana and across SSA. This analysis only captures a snapshot of attitudes reflected during data collection (2017) and may not reflect current attitudes given ongoing program development. Future analysis is needed to look for conservation of these attitudes. Lastly, this analysis does not include maternal mortality data. This project is an ongoing process rather than an impact project, and as such, aims to discover a process for increasing Ob/Gyn capacity with the implication of reducing maternal mortality.

## Conclusion

With over 30 years of training, development, and retention, the Ghana Ob/Gyn training program is effectively filling the gap in obstetric capacity by completing the maternal care team and its ability to provide contemporary comprehensive obstetric care. The program has a unique perspective on the strengths and barriers to specialty training in low-resource settings. It has the potential to serve as a model for international academic medicine partnerships and can be adapted to other low-resource settings. The program has demonstrated efficacy in increasing national maternal health and obstetric capacity and is on the brink of drastically increasing women’s health capacity, reducing preventable maternal mortality, and driving SDG progress with the growth of the Ob/Gyn generalist population and the projected growth of subspecialist population. This additional data analysis served to further study this model to contribute to the program’s development, reproducibility, and applicability to other medical specialties.

## Supporting information

S1 Checklist COREQ (COnsolidated criteria for REporting Qualitative research) checklist. (PDF)

S1 Questionnaire PLOS ‘inclusivity in global research’ questionnaire. (DOCX)
